# Validation of the German Version of Functional Oral Intake Scale (FOIS-G) for Flexible Endoscopic Evaluation of Swallowing (FEES)

**DOI:** 10.1007/s00455-020-10114-1

**Published:** 2020-04-27

**Authors:** Samra Hamzic, Tobias Braun, Martin Juenemann, Marius Butz, Robert Voswinckel, Michael Belly, Oliver Vogelbusch, Susanne Weber, Hasan Khilan, Manfred Kaps, Tibo Gerriets

**Affiliations:** 1 Department of Neurology, University Hospital Giessen and Marburg GmbH, Klinikstrasse 33, 35392 Giessen, Hesse Germany; 2 Buergerhospital Friedberg (Hesse), Stroke Unit, Ockstaedter Str. 3-5, 61169 Friedberg, Hesse Germany

**Keywords:** Dysphagia, Deglutition, Deglutition disorders, FOIS, Cross-cultural adaptation, FEES, German version, Validation

## Abstract

The Functional Oral Intake Scale (FOIS) is the most frequently used scale for the evaluation of functional oral intake by dysphagia patients. FOIS was validated using data from Videofluoroscopic Swallowing Study (VFSS). Until now, a validated German version of FOIS for Flexible Endoscopic Evaluation of Swallowing (FEES) is lacking. The aim of this study was a cross-cultural validation of the German version of FOIS (FOIS-G) for FEES. The translation of the original FOIS was carried out according to the Translation, Review, Adjudication, Pretesting, Documentation (TRAPD) translation methodology. For the validation process, six experienced language therapists (SLT) retrospectively analyzed charts of 93 stroke patients. Inclusion criteria were comprised of stroke, clinical examination by an SLT within 24 h of admission, and FEES within 72 h of admission. The validity was calculated by comparison with Modified Rankin Scale (MRS), Barthel Index (BI), the Penetration-Aspiration-Scale (PAS), and a water swallow test. Spearman rank correlation of all paired raters ranged from *r*_s_ = 0.96 to *r*_s_ = 0.99, and percentage agreement ranged from 81 to 94%. The overall agreement between all raters was calculated by Fleiss kappa (0.83) (s.e. 0.02). There is a significant correlation between the BI and the MRS with the FOIS-G (*r*_s_ = 0.301, *p* = 0.003 for BI; *r*_*s*_ = – 0.366, *p* < 0.001 for MRS), between the PAS and the FOIS-G (*r*_s_ = − 0.758, *p* < 0.001), as well as between the 70 ml-water-test and the FOIS-G (*r*_s_ = 0.470, *p* < 0.001). FOIS-G is a valid instrument for the evaluation of the functional oral intake of food and liquids in dysphagia patients.

## Introduction

Neurogenic dysphagia comprises of a disordered intake of fluids and food due to neurologic diseases. It causes restrictions in patients’ oral ability to intake and process secretions, food, and fluids. Dysphagia may be a cause of malnutrition, dehydration, and aspiration pneumonia and can entail a prolonged length of hospital stay. As a consequence, patients may encounter long-term artificial nutrition, invasive ventilation via tracheotomy tubes, reduced quality of life, and, lastly, death [1–4].

Dysphagia is a common consequence of a stroke. Its incidence among stroke survivors shows a high degree of variability ranging from 19 to 81% when imaging methods for dysphagia like Videofluoroscopic Swallowing Study (VFSS) or Flexible Endoscopic Evaluation of Swallowing (FEES) are implemented [1, 5–8]. Six months after stroke, up to 50% of patients still suffer dysphagia [9, 10].

Early detection of dysphagia is beneficial for the overall outcome by reducing the risks of mortality and of secondary complications such as aspiration pneumonia, dehydration, and malnutrition as well as the length of hospitalization and the overall costs of treatment [11].

An adequate care of dysphagia patients includes the application of validated clinical and instrumental diagnostic methods and scales. An imaging method, FEES, has become the gold standard in Germany and has been implemented in more than 70% of stroke units [12]. The scores most frequently used for objective evaluation of dysphagia severity are the Penetration-Aspiration-Scale (PAS) [13], the Secretion Severity Rating Scale (SSRS) [14] and the FOIS scale [15]. These scores allow for monitoring of swallowing ability and security over time.

The functional oral intake scale (FOIS) was developed in 2005 as a tool with very good reliability, validity, and sensitivity to change to objectively determine and monitor the range of oral intake of patients with neurogenic dysphagia [15]. It is an ordinal scale with seven tiers that assesses the oral intake of food and liquids. Different ranges of non-oral feeding are subsumed in levels 1–3, whereas different ranges of oral feeding are included in levels 4–7. It has been the most commonly used scale for the rating of the range of oral intake by patients suffering from dysphagia and is used both in clinical and in research settings [16, 17] as well as in various patient populations (patients with amyotrophic lateral sclerosis, head and neck cancer, Parkinson’s disease, and pediatric patients) [18–21].

Functional rating scales have been applied as assessment protocols, tools for evaluation of patient outcomes and for detection of changes in swallowing over time [22]. Furthermore, they can be used to monitor the adequacy and effectivity of training and rehabilitation methods. Compared to Functional Outcome Swallowing Scale (FOSS) [23], the Food Intake Level Scale (FILS) [24] and the Dysphagia Outcome and Severity Scale (DOSS) [25], each lacking either reliability, validity, or sensitivity to change over time, FOIS is an impairment-specific scale with precisely defined differences between easily understood scale levels and an excellent psychometric quality. In our experience and as confirmed in various studies [16–21], FOIS has shown to be an excellent and very practical tool for assessing functional oral intake in dysphagic patients and for monitoring rehabilitation achievements over time. We are committed to the use of best validated procedures and scores for our patients to monitor the range of oral intake and the efficacy of both dysphagia and nutritional treatment. The lack of a uniform worldwide approach and guidelines for patient-oriented, time- and cost-effective dysphagia management is a well-known fact [26, 27]. The implementation of validated scales like FOIS in several languages is an important step in this direction and paves the way to maximize comparability of international research. The aim of this study is to satisfy these demands for the German language and to conduct a cross-cultural validation of the German version of the FOIS scale (FOIS-G).

For the translation process, we implemented the TRAPD-procedures (Translation, Review, Adjudication, Pretesting and Documentation) and a committee-based approach to translation process, which does not include the back translation methodology [28].

The TRAPD-method as a five-step team-based approach suggests parallel translation of the source text in cooperation between three different sets of persons: translators, reviewers, and adjudicators [29]. All members own a mixture of skills and expertise allowing for an optimal decision on the best version. The team has a profound knowledge of the study issue, the measures to be translated, and the underlying research topic. Finally, all team members need to possess a high level of linguistic and cultural knowledge in order to establish an adequate version in the target language [29–34]. According to the TRAPD-method, more than one translator is needed for the translation from the source into the target language. At least one person, who is experienced in the principles of questionnaires and surveys design, linguistics, and translations, is also included in the reviewing process. The adjudicator is specialized in the research topic, having knowledge of both the target and the original language and is in charge of all final decisions concerning the final translation version and can take the role both of reviewer and adjudicator (“reviewer cum adjudicator”) [29].

## Materials and Methods

### Translation Process According to TRAPD-Methodology

#### Phase 1: Translation

For the forward translation from English into German the parallel translation method was selected: two neurologists and a speech and language therapist (SLT), who are active in dysphagia research and have a proficient and fluent command of written and spoken English, produced independently parallel translation drafts.

#### Phase 2: Review

The review of the forward translation drafts was assigned to the translators and two reviewers (first author of this article being one of them). The goal of the review step was to identify discrepancies and special difficulties between the original scale and the three parallel translations and decide on a preliminary version of FOIS-G.

#### Phase 3: Adjudication

In a joint expert panel, all persons included in the forward translation and the review process discussed the final version of FOIS-G to be adopted. For this expert panel, the author of this article was in charge as reviewer cum adjudicator [29] and made the final decision on the final consensus of FOIS-G version, which was used for pretesting and validation (Table 1).

**Table 1:** The original version of the Functional Oral Intake Scale (FOIS) and the German version (FOIS-G)

Functional Oral Intake Scale (FOIS)
Level 1: Nothing by mouth Level 2: Tube dependent with minimal attempts of food or liquid Level 3: Tube dependent with consistent oral intake of food or liquid
Level 4: Total oral diet of a single consistency Level 5: Total oral diet with multiple consistencies but requiring special preparation or compensations Level 6: Total oral diet with multiple consistencies without special preparation, but with specific food limitations Level 7: Total oral diet with no restrictions
Functional Oral Intake Scale in German (FOIS-G)
Stufe 1: Keine orale Ernährung Stufe 2: Sondenabhängig mit minimalen Versuchen oraler Nahrungs- oder Flüssigkeitsaufnahme Stufe 3: Sondenabhängig mit regelmäßiger oraler Nahrungs- oder Flüssigkeitsaufnahme
Stufe 4: Vollständige orale Aufnahme einer Nahrungsmittelkonsistenz Stufe 5: Vollständige orale Aufnahme mehrerer Nahrungsmittelkonsistenzen; spezielle Zubereitung oder Kompensation erforderlich Stufe 6: Vollständige orale Aufnahme mehrerer Nahrungsmittelkonsistenzen ohne spezielle Zubereitung; Einschränkung bestimmter Nahrungsmittel erforderlich Stufe 7: Vollständige orale Nahrungsaufnahme ohne Einschränkungen

#### Phase 4: Pretesting

Pretesting checks for explicit comprehension, routing, and other implementation problems. The pretesting of FOIS-G was carried out by presenting a list of 114 oral diet recommendations after FEES to two SLTs with expertise in FEES, dysphagia treatment, and research. Both assigned a FOIS-G score to each of 114 oral diet recommendations independent of each other.

#### Phase 5: Documentation

**Fig. 1:**
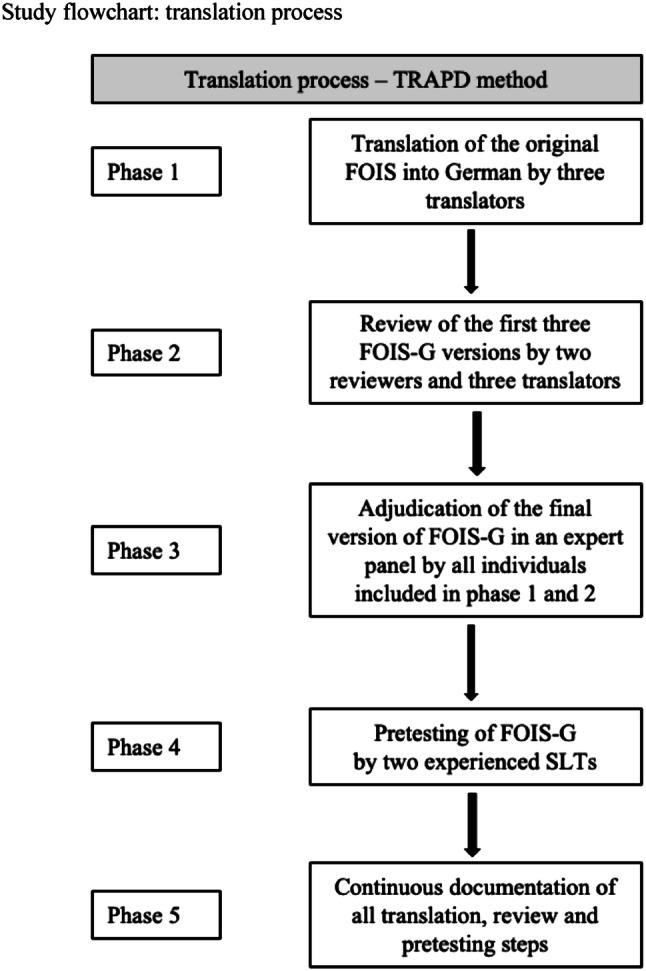
Study flowchart: translation process

The entire TRAPD-process is accompanied by a continuous documentation of all steps, review and expert panels (draft translations, exchange of notes between the translators, the reviewers, and the adjudicator, pretesting results and exchange of comments between the SLTs involved, notes on final translation). Notes and documentation from previous steps are necessary information tools for ongoing phases and build a basis for decisions in the next steps (Fig. 1).

## Validation Process

### Study Design

We perceived the validation of FOIS-G as an important step towards the further optimization of clinical dysphagia management. Therefore, a retrospective design for this study was chosen since all data needed to conduct the validation of FOIS-G were already available in the in-house hospital documentation system. This design allowed for a time- and cost-effective study implementation and data analysis.

**Fig. 2:**
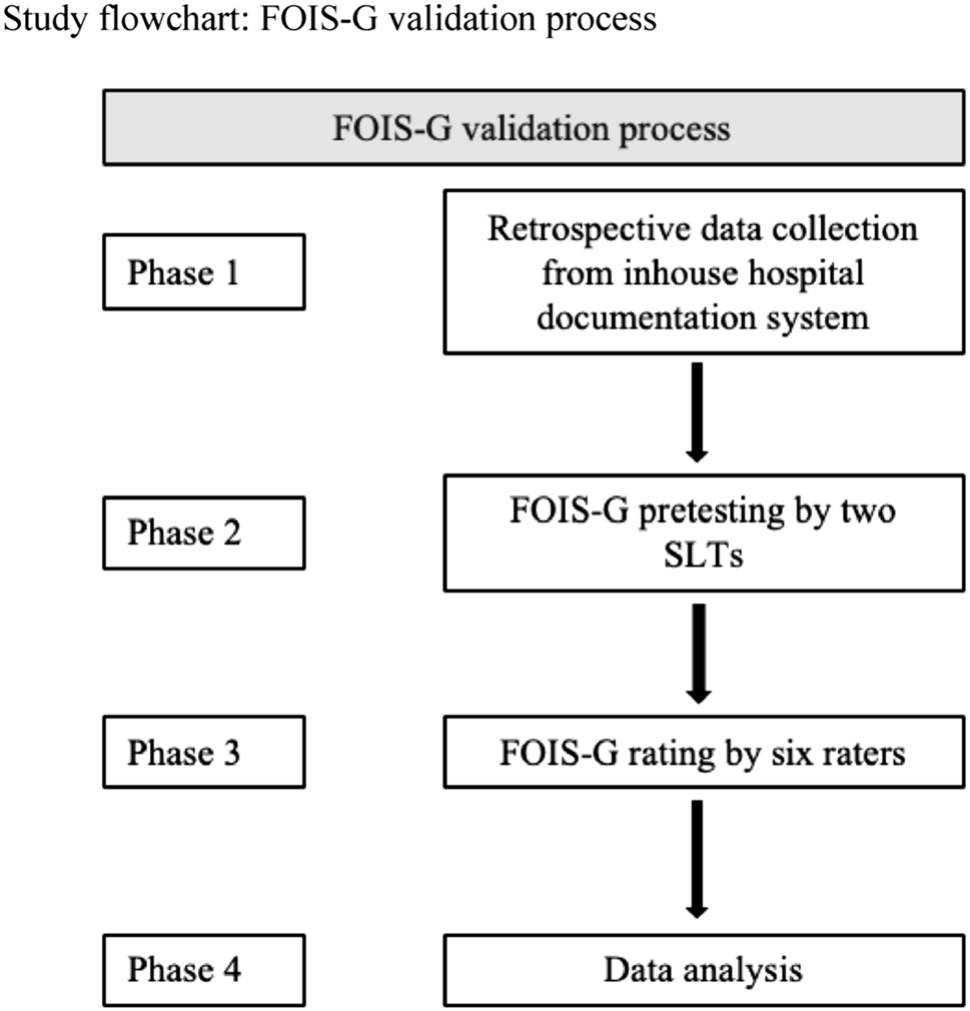
Study flowchart: FOIS-G validation process

For the pretesting and the validation process, a retrospective evaluation of clinical charts of stroke patients administered to the stroke unit at the community hospital in Friedberg, Germany, between January 2015 and December 2017 was conducted (Fig. 2).

### Inclusion and Exclusion Criteria

We revised a total of 93 patient charts who were consecutively administered to the stroke unit and who met the inclusion criteria of (1) ischemic stroke as diagnosed per a cranial computed tomography (CT) or a magnetic resonance imaging (MRI), (2) standard stroke treatment according to the stroke guidelines of the German Association of Neurology, (3) scoring for Modified Rankin Scale (MRS) and Barthel Index (BI), and (4) clinical examination as well as the 70 ml-water-test by an SLT within 24 h of admission, (5) FEES within 72 h of admission. 11 of 93 patients had two FEES and 5 of 93 patients had three FEES within 2 to 13 days after admission. A total of 114 oral diet recommendations for 93 patients after FEES was included for the validation of FOIS-G.

Patients who were administered to the stroke unit but did not undergo a FEES examination were excluded from the study.

### Data Collection

The data gathering for the cross-cultural adaptation of FOIS in German was based on the study design of the original work [15]. However, some measures varied due to cross-cultural differences in implementation of stroke guidelines. In the original work, the following measures were compared for the validation process: Modified Rankin Scale (MRS), the Modified Barthel Index (MBI), and Mann Assessment of Swallowing Ability (MASA). Finally, all patients underwent a VFSS within 72 h of admission in which the severity of dysphagia and aspiration presence and severity was assessed.

The MRS, as a 7-tiered scale, is a functional outcome measure for stroke patients measuring the level of disability, whereas MBI scores the dependence of stroke survivors in activities of daily living after stroke. In clinical trials, MRS and MBI are frequently implemented as primary outcome measures.

MASA is a bedside screening tool to detect swallowing disorders and aspiration in acute stroke patients showing significant sensitivity and specificity [35–37]. However, the clinical screening tool for dysphagia most frequently used in German stroke units is the water-test according to Daniels (further referring to as 70 ml-water-test), which shows a 93% rate of sensitivity and a 67% rate of specificity in detecting aspiration risk in acute stroke patients [38].

In contrast to VFSS, which is the gold standard of imaging diagnostics in the United States, FEES is the method of choice in Germany [12].

**Fig. 3:**
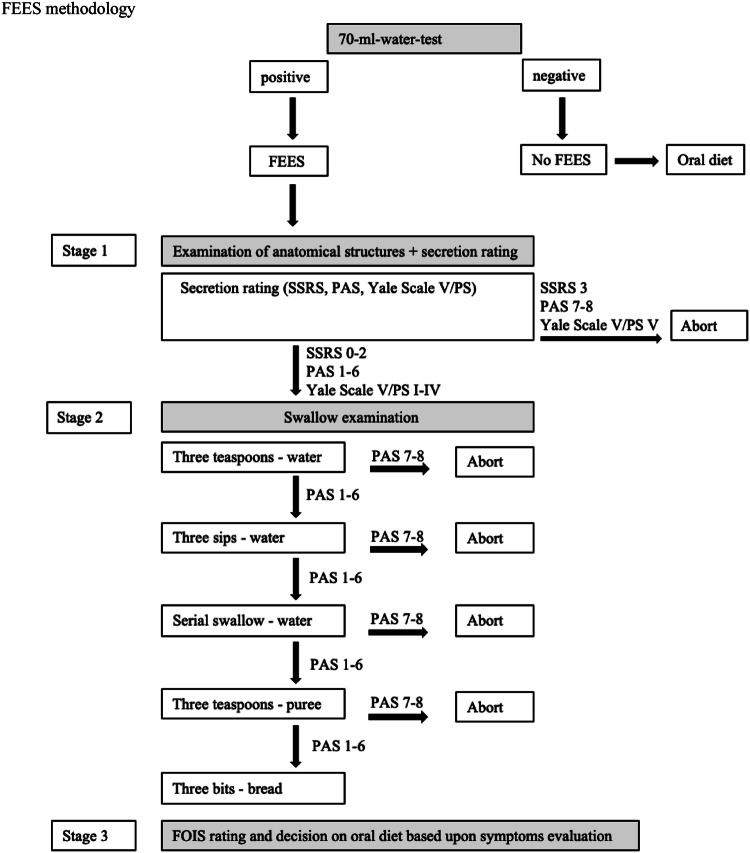
FEES methodology. *SSRS* secretion severity rating scale, *PAS* penetration- aspiration-scale, *Yale Scale V/PS* yale pharyngeal residue severity rating scale, *V* valleculae, *PS* Piriform sinus)

For our validation study, we used following outcome measures, which are commonly assessed in German stroke units: the MRS, the standard Barthel Index (BI), the 70 ml-water-test and the PAS score for FEES (Fig. 3, Table 2).

**Table 2:** Clinical and demographic features of 93 stroke patients

Demographic and clinical features	*n* = 93	FOIS ratings after initial FEES						
		1	2	3	4	5	6	7
		22	3	8	1	27	9	23
Mean age ± SD (years)	77.85 ± 10.55	80.18 ± 7.16	82.33 ± 13.70	80.62 ± 8.93	83 ± 0	81.30 ± 7.42	73.11 ± 15.23	71.65 ± 10.96
Sex (%)								
Male	64.52	77	33	50	100	59	67	65
Female	35.48	23	67	50	0	41	33	36
Pathology								
Cerebral infarction	76	20	3	6	0	22	7	18
Cerebral hemorrhage	13	2	0	0	1	5	2	3
Transient ischemic attack	4	0	0	2	0	0	0	2
Mean BI score	32.47	21.81	8.33	12.5	10	32.22	51.11	46.74
Mean MRS score	3.49	3.95	4.33	4.25	5	3.37	3.22	2.87
Mean PAS score	3.85	6.81	2	5	2	4.37	1.78	1.13

*BI* Barthel Index, *MRS* Modified Ranking Scale, *PAS* Penetration-Aspiration-Scale

### FEES Methodology

The clinician performing FEES is an experienced SLT and dysphagia therapist and holder of the FEES Instructor Certificate of the German Society of Neurology and the European Society for Swallowing Disorders with more than ten years of experience in FEES in conducting evaluation and research. The FEES examination is carried out in three sections: (1) examination of anatomical structures and secretion rating, (2) swallow examination, and (3) symptoms evaluation. Validated scales are used for the evaluation of swallowing: The Secretion Severity Rating Scale (SSRS) [14], the Penetration-Aspiration-Scale (PAS) [13], and the Yale Pharyngeal Residue Severity Rating Scale for Valleculae and Piriform sinus (Yale Scale V/PS) [39]. Exactly defined amounts of liquid (1 teaspoon = 3 ml; 1 sip = 10 ml), pureed (1 teaspoon = 4 ml), and solid boluses (5 g) are administered each three times to the patients. Following cutoff values of the scales for saliva (SSRS = 3, PAS ≥ 7, Yale Scale Valleculae /Piriform sinus = V), liquid ((PAS ≥ 7), pureed (PAS ≥ 7), and solid boluses (PAS ≥ 7) determine when to abort the swallow examination. Finally, the FOIS scale and oral diet are recorded after the evaluation of swallowing capacity on the basis of perceived symptoms and determined scale scores.

### FOIS-G Pretesting

114 oral diet recommendations built the basis for FOIS-G pretesting by two experienced SLTs with more than 10 years of experience in FEES and dysphagia management. In the first step, a FOIS-G score was assigned to each diet recommendation separate from each other. In a subsequent joint discussion, the assigned scores were discussed by the two SLTs and in cases of deviations a mutual compromise was determined. The agreement between the two SLTs served as the gold standard for validity analysis as well as for the following ratings by six experienced SLTs.

### FOIS-G Rating

Six dysphagia experienced SLTs with German as their native language working at various hospital sites in Germany and Austria were recruited for the rating of FOIS-G. Their working experience ranged from 2–19 years (mean 10.5 years). The sole training in the usage of the FOIS-G was the presentation of the scale a week before the actual rating took place. The raters had one week to ask questions and discuss the usage of the scale with the author of this article. All SLTs were blinded about each other’s ratings and the pretesting of FOIS-G. For the rating of FOIS-G, the participating SLTs were asked to assign a FOIS-G score to the 114 oral recommendations. A total of 100% of SLTs has conducted the rating. The evaluation of six paired raters were all blinded to each other.

### Statistical Analysis

Statistical analyses were performed using SPSS 25.0 statistical software (IBM, SPSS, Inc., Chicago, IL, USA). The calculation of Fleiss kappa and linear weighted Cohen’s kappa were carried out with Real Statistics Resource Pack (www.real-statistics.com), a free add-in software for Microsoft Excel.

### Inter-Rater Reliability

Due to the possible agreement between the two SLT’s by chance, we used Cohen’s kappa statistic, especially a linear weighted Cohen’s kappa to attribute more weight on higher disagreements [40]. To calculate inter-rater reliability between paired raters we used percentage agreement and Spearman rank correlation. In addition, to determine the overall agreement between all six raters by subtracting out agreement due to chance, we used Fleiss kappa [40]. 

### Criterion Validity

To evaluate criterion validity, the association between the FOIS-G ratings and BI and MRS was calculated with Spearman rank correlation. Furthermore, we dichotomized the data from BI and MRS with established criteria (MRS score ≤ 3, BI ≤ 75) and used *χ*^2^ and Cramer’s V statistic for comparisons. In the original work, the dichotomization was set at ≤ 3 for MRS and ≤ 15 for MBI for moderate disability. For our validation, we set the dichotomization of BI at ≤ 75, which usually represents moderate disability according to Geert et al. 1999 [33]. We expected significant positive correlation of the FOIS with BI and a significant negative correlation of the FOIS with MRS both for dichotomized and non-dichotomized data.

### Cross-Validation

Cross-validation between FOIS-G ratings and PAS as well as between FOIS-G ratings and the 70 ml-water-test was calculated with Spearman rank correlation. We expected significant negative correlation of the FOIS-G with the PAS score and a significant positive correlation of the FOIS-G with the 70 ml-water-test.

## Results

### Inter-Rater Reliability

The agreement of the two SLT’s during the pretesting, which was calculated with linear weighted Cohen’s kappa, was high (*κ* = 0.96, s.e. 0.02). Percentage agreement between all paired raters ranged from 81 to 94%. Spearman rank correlation of all paired raters ranged from 0.96 to 0.99. The overall agreement between all six raters by using Fleiss kappa was high (*κ* = 0.83, s.e. 0.01) (Tables 3 and 4).

**Table 3:** *χ*^2^, Cramer’s V, and Spearman’s rho Correlation between the FOIS-G (pretesting) and the BI and MRS scores taken at pre-admission and admission to stroke unit and discharge from stroke unit

Test	*χ*^2^	*p*	Cramer’s V correlation	Spearman rho	*p*
Pre-admission					
MRS	8.887	0.180	0.309	− 0.329	0.001
Admission					
BI	4.376	0.626	0.217	0.301	0.003
MRS	12.213	0.057	0.362	− 0.366	< 0.001
Discharge					
BI	11.803	0.067	0.356	0.520	< 0.001
MRS	18.563	0.005	0.447	− 0.474	< 0.001

**Table 4:** *χ*^2^, Cramer’s V and Spearman`s rho correlation between the FOIS-G (average six raters) and the BI and MRS Scores taken at pre-admission and admission to stroke unit and discharge from stroke unit

Test	*χ*^2^	*p*	Cramer’s V correlation	Spearman rho	*p*
Pre-admission					
MRS	17.165	0.579	0.430	− 0.344	0.001
Admission					
BI	15.944	0.661	0.414	0.307	0.003
MRS	23.843	0.202	0.506	− 0.389	< 0.001
Discharge					
BI	21.072	0.333	0.476	0.523	< 0.001
MRS	30.992	0.040	0.577	− 0.497	< 0.001

### Criterion Validity

Spearman rank correlation reveals that all stroke measures (MRS, BI) were significantly correlated with FOIS-G (pretesting) score and FOIS-G (average six raters) score on pre-admission, admission to stroke unit, and discharge from stroke unit (Tables 3 and 4).

*χ*^2^ calculation of dichotomized data shows significant associations between FOIS-G (pretesting) score and MRS at discharge from stroke unit (*χ*^2^ = 18.563, *p* = 0.005). FOIS-G (average six raters) score was significantly associated with MRS at discharge from stroke unit (*χ*^2^ = 30.992, *p* = 0.040). With dichotomized data, no association was found between FOIS-G scores and BI (Tables 3 and 4).

### Cross-Validation

Spearman rank correlations reveals that the PAS score is significantly correlated with FOIS-G (pretesting) score (*r*_s_ = − 0.758, *p* < 0.001) and FOIS-G (average six raters) score (*r*_s_ = − 0.757, *p* < 0.001).

The 70 ml-water-test could not be performed on all patients due to various post-stroke symptoms such as impaired vigilance, aphasia or speech apraxia. Therefore, we calculated the Spearman rank correlation between FOIS-G scores and 70 ml-water-test scores with a sub sample size of 76 subjects. We found significant correlations between 70 ml-water-test scores and FOIS-G (pretesting) score (*r*_s_ = 0.542, *p* < 0.001) and FOIS-G (average six raters) score (*r*_s_ = 0.534, *p* < 0.001).

## Discussion

The FOIS has been the most commonly used scale for the rating of the range of oral intake by patients suffering dysphagia and is used both in clinical and in research settings.

This retrospective study aimed at cross-cultural adaptation of the FOIS scale into German (FOIS-G). We conducted the translation by implementing the team approach within the TRAPD-methodology without the interim step of back translation. Even though usually implemented and recommended in cross-cultural adaptation processes of surveys, questionnaires, and self-reported outcome measures [42, 43], the method of back translation, first, does not possess a profound science-based background and, second, does not always ensure an improved quality of the final version [31, 32].

The validation process was based on the study design of the original scale. Not all items used in the original work were included in the validation of the German version due to cross-cultural differences in the implementation of stroke treatment guidelines. The inter-rater reliability was high for both the pretesting by two experienced SLTs and for the rating by six experienced SLTs and presented a significant correlation between all included stroke measures and the FOIS-G. The minimal discrepancy between the two SLTs in pretesting (in only three cases) was due to insecurities concerning the definition of oral intake of patients with regular oral intake parallel to intravenous nutrition. After consultation with the original FOIS author, it was determined that intravenous nutrition is equal to tube-dependent intake.

As expected, we found significant statistical correlations between the FOIS-G and all outcome measures: the MRS, the BI, the PAS score and with the 70 ml-water-test. These results are very similar to the original work despite not fully equal study designs. The FOIS-G inter-rater reliability with *K* = 0.96 between two raters for pretesting as well as the percentage agreement for all six paired raters are high with 81% to 94% for FOIS-G vs. 85% to 95% for the original FOIS. Spearman rank correlation between all raters in FOIS-G is *r*_s_ = 0.96 to *r*_s_ = 0.99 (original FOIS *r*_s_ = 0.98 to *r*_s_ = 0.99). Overall agreement between all six paired raters for FOIS-G is summed up to *K* = 0.83 (original FOIS *K* = 0.86 to *K* = 0.91).

As for criterion validity without dichotomization the stroke measures (MRS, BI, and 70 ml-water-test) correlated significantly with FOIS-G both in pretesting as well as in the evaluation by six paired raters on pre-admission, at admission and at discharge from stroke unit. As in the original work our dichotomized data for MRS BI and 70 ml-water-test show a significant association between FOIS-G and MRS at discharge both for pretesting as well as for six average raters. Equivalent to the results of cross-validation of the original FOIS and VFSS a significant correlation between FOIS-G and the PAS score in FEES was found both in pretesting with two raters as well as in rating by all six paired raters. The 70.-ml-water-test in a subsample of 76 subjects shows a significant correlation with FOIS-G.

Even though the cross-cultural adaptation of the Chinese and the Italian version of the FOIS have been conducted in different study settings very strong similarities are found with those results as well: The inter-rater reliability for both Chinese and Italian version are strong (Italian FOIS ICC = 0.99; Chinese FOIS *K* = 0.881, Spearman rank correlation *r*_s_ = 0.972; Chinese water swallow test *K* = 0.844, Spearman rank correlation *r*_s_ = 0.965). The Italian FOIS version did not conduct the calculation of criterion validity nor of cross-validation. However, the Chinese results are very similar to the original FOIS and the FOIS-G with a strong correlation found between FOIS and the water swallow test. Furthermore, NIHSS and MBI are also significantly associated with the Chinese FOIS. Cross-validation shows a high association with the Chinese FOIS and the presence of dysphagia and aspiration in VFSS.

Despite the different approaches of the cross-cultural adaptions of the original FOIS scale it is recognizable that all three validated translations show a high inter-rater reliability and, except the Italian version, very strong correlations for criterion validity and cross-validation.

The different study designs are due to the fact that there is no uniform approach to dysphagia management worldwide. In Germany and Italy, the water swallow test is carried out by SLTs, in China by nurses. In addition, there is still no worldwide consensus on which clinical swallow test whether FEES or VFSS should be used as gold standard of instrumental dysphagia diagnostic tool. As consequence, FEES and VFSS are not uniformly used in the same quantity and quality as the gold standard for instrumental dysphagia diagnostics.

The similarity in inter-rater reliability between all three translated versions of the original FOIS is due to the good consensual and criterion validity of the original FOIS scale. As for the cross-validation the fact that the results for both VFSS and FEES are similar in all three translated versions shows that both instrumental tools as well as the FOIS mirror a high validity.

In Germany FEES has become the gold standard of instrumental dysphagia diagnostics being used in more than 70% stroke units [12], whereas VFSS is found only in a few facilities across the country. Validating FOIS-G for FEES adds both to the value of the FOIS and to the FEES examination in research and clinical settings. The results of this study consolidate FEES as an important diagnostic tool in the acute stroke setting as well as in the acute stroke dysphagia management and show the relevance of the implementation of FOIS in everyday clinical practice.

## Study Limitations

We did not conduct the inter-rater reliability of FOIS-G for the 70 ml-water-test since at that point of time the FOIS was recorded only for FEES data. This clearly is a limitation to this study as well as the retrospective design of the study. The retrospective characteristic of the study may have caused the negative correlation between the FOIS-G and the BI in dichotomized data contrary to the original work where all stroke measures show a strong association with FOIS in criterion validity for dichotomized data. It is presumable that raising the BI score in a prospective study design prior to the FEES may have resulted in positive results for the correlation between FOIS-G and BI.

With the increasing globalization of the evidence-based medicine, we see, in an ideal case, a uniform description of the results concerning both the transnational clinical patient care and research. Scores are an opportunity to enable and establish international comparability. However, in this context, a thorough validation of each test in the language of each country is an obligatory/irrefutable condition. In the case of the FOIS, the German version, at hand, is only the third translation (besides the Italian and Chinese version [44, 45]) from the 2005 original scale published in English. This circumstance emphasizes, on the one hand, the need for additional, comprehensive translations and validations and, on the other hand, the simultaneous development of new scales in a variety of languages with nominal time, economic and personal associated investments. Along these lines, we support future structural efforts towards a change in paradigm by means of international cooperation in the development of new dysphagia scores and/or the modification of already existent scales.

The design and results of the present study as well as the comparison with existing adaptations show the necessity of a worldwide uniform approach in the design of dysphagia management. The use of validated scales in several languages is an important step in this direction.

## Conclusion

FOIS-G was translated according to international translation guidelines and validated by experienced SLTs with German as their native language. It is a valid instrument for the evaluation of functional oral intake of liquids and food by dysphagia patients and can be easily implemented both in clinical and research settings.
